# Using LongSAGE to Detect Biomarkers of Cervical Cancer Potentially Amenable to Optical Contrast Agent Labelling

**Published:** 2007-12-11

**Authors:** Julie M. Kneller, Thomas Ehlen, Jasenka P. Matisic, Dianne Miller, Dirk Van Niekerk, Wan L. Lam, Marco Marra, Rebecca Richards-Kortum, Michelle Follen, Calum MacAulay, Steven J. M. Jones

**Affiliations:** 1 Genome Sciences Centre, British Columbia Cancer Research Centre, Vancouver, BC, Canada; 2 Department of Gynaecologic Oncology, British Columbia Cancer Agency, Vancouver, BC, Canada; 3 Cancer Imaging, British Columbia Cancer Research Centre, Vancouver, BC, Canada; 4 Cervical Cancer Screening Program, British Columbia Cancer Agency, Vancouver, BC, Canada; 5 Cancer Genetics and Developmental Biology, British Columbia Cancer Research Centre, Vancouver, BC, Canada; 6 Biomedical Engineering, University of Texas at Austin, Austin, TX, U.S.A; 7 University of Texas M.D. Anderson Cancer Center, Department of Gynecologic Oncology and Biomedical Engineering Center, Houston, TX, U.S.A

**Keywords:** longSAGE, cervical cancer, biomarker, optical imaging

## Abstract

Sixteen longSAGE libraries from four different clinical stages of cervical intraepithelial neoplasia have enabled us to identify novel cell-surface biomarkers indicative of CIN stage. By comparing gene expression profiles of cervical tissue at early and advanced stages of CIN, several genes are identified to be novel genetic markers. We present fifty-six cell-surface gene products differentially expressed during progression of CIN. These cell surface proteins are being examined to establish their capacity for optical contrast agent binding. Contrast agent visualization will allow real-time assessment of the physiological state of the disease process bringing vast benefit to cancer care. The data discussed in this publication have been submitted to NCBIs Gene Expression Omnibus (GEO, http://www.ncbi.nlm.nih.gov/geo/) and are accessible through GEO Series accession number GSE6252.

## Introduction

Clinical diagnosis of most cancers and their precursors is predominantly based on phenotypic markers such as appearance of cell nuclei. Classification and staging of disease is determined by evaluation of gross structural features, such as extent of local tumour invasion and presence of disease in other organs. It is now established that cancer arises as a result of successive genetic changes altering cellular processes including growth, angiogenesis, senescence, and apoptosis (Hanahan and Weinburg, 2000). Additionally, many cancers appear to have active inflammation and wound healing mechanisms (Chang et al. 2004). Proteins taking part in these cellular mechanisms are often strong candidates for biomarkers and molecular targets.

Cervical cancer is usually the result of a human papillomavirus (HPV) infection which initiates neoplastic progression mainly through viral oncoproteins E6 and E7 within the cervical transformation zone at the squamous/columnar junction. The role of HPV to the pathogenesis of cervical cancer has been addressed in recent reviews (zur Hausen, 2002; Woodman et al. 2007). Many HPV types produce only productive lesions following infection and are not associated with human cancers. In such lesions, the expression of viral gene products is carefully regulated, with viral proteins being produced at defined times and at regulated levels as the infected cell migrates towards the epithelial surface. The events that lead to viral synthesis in the upper epithelial layers appear common to both the low- and high-risk HPV types. Virus-induced cancers most often arise at sites where productive infection cannot be suitably supported. Productive infection can be divided into distinct phases, with different viral proteins playing specific roles (Doorbar, 2006). Upon infection, normal cells gradually advance through stages of cervical intraepithelial neoplasia (CIN). Mild dysplasia (CINI) presents as only a subset of the low third of the epithelium appearing dysplastic, moderate dysplasia (CINII) occurs where the dysplastic cells involve about one-half of the thickness of the epithelium of the cervix, and severe dysplasia (CINIII), or carcinoma-in-situ, is described as the condition where the entire thickness of the epithelium is disordered but the abnormal cells have not yet spread below the surface. If carcinoma-in-situ is not treated, it will often grow into an invasive cervical cancer. High grade dysplasia is considered the most advanced dysplasia with atypical changes in many of the cells and a very abnormal growth pattern of the glands; some of the glands are branching or budding. More than 50% of the cells have large, spotted nuclei and are frequently dividing while the cellular cytoplasm is reduced and looks abnormal. Cancer of the cervix was one of the most common causes of cancer death for American women, but between 1955 and 1992 the number of cervical cancer deaths in the United States dropped by 74% due to the introduction of the Pap test (Papanicolaou and Traut, 1943). Death rates from cervical cancer continue to decline by nearly 4% per year. Even so, the American Cancer Society reports that in 2006, about 3,700 of the 9,710 women diagnosed with cervical cancer in the United States have died from this disease.

HPV infection causes changes in expression levels of a wide variety of genes (Yim and Park, 2006). These differences in gene expression between pre-invasive neoplastic and non-neoplastic tissue give clues to the molecular basis of cancer. Early detection of cervical cancer based on molecular characterization would be clinically advantageous; risk of neoplastic lesion progression could be predicted and response to therapy could be monitored in real time at a molecular level. To monitor molecular characterisation of cancer it follows that the ability to optically image in real-time the molecular features of cancer *in vivo* is critical (González et al. 1999; Rajadhyaksha et al. 1999; White et al. 1999; Huzaira et al. 2001; Langley et al. 2001; Selkin et al. 2001; Collier et al. 2002) and requires safe, molecular-specific contrast agents whose images can be monitored rapidly and non-invasively during their uptake and distribution. The analysis presented here evaluates serial analysis of gene expression (SAGE) libraries to identify novel, cell-surface gene products. Upon mapping of highly differentially expressed SAGE tags to their corresponding genes, the gene products are candidates for antibody testing and optical contrast agent development.

## Contrast Agents and Optical Imaging

Short of prevention, improved early stage cancer diagnosis would provide the greatest benefit for cancer patients. Because proteins may regulate gene expression, ligand-binding properties, molecular structure and dynamics on a temporal basis, protein biomarkers have a significant impact in cancer detection and therapy as therapies are becoming targeted to specific signal transduction and metabolic pathways. For example, breast cancers respond to HERCEPTIN (trastuzumab) if the tumor over-expresses Her-2/neu (Baselga et al. 2004; Ross et al. 2004). In the same way, GLEEVEC (imatinib) is most effective against cancers carrying the bcr-Abl translocation (Druker, 2004) and targeted molecular cancer therapy is already used successfully for the eradication of acute leukaemia (Frater et al. 2003; Yee and Keating, 2003). These examples imply that it will be important to produce biomarkers for all stages of cancer. Reliable diagnostics such as DNA screening and immunocytochemical analysis of known cervical neoplasia biomarkers p16^INK4A^ and minichromosome maintenance (MCM) proteins are not implemented *in vivo*. Real-time biomarkers of the physiological state of the disease process or markers representative of treatment efficacy will bring immeasurable benefit to cancer care in terms of individualized agent selection and dosing. Furthermore, series of agents could be tested to determine empirically the localization of cancer and/or the most effective therapy.

Routine clinical cancer detection employs non-specific contrast agents such as acetic acid which enhance the nuclear backscattering but are limited by small signal magnitude. The field of molecular imaging is rapidly developing imaging agents with high affinity and specificity for targeted biomarkers. These new agents allow for the possibility of disease detection earlier than is currently feasible (Weissleder, 2001; Jaffer and Weissleder, 2005). For example, cancer metastases missed by conventional anatomically based imaging methods may be detected in patients by molecular imaging (Harisinghani et al. 2003). Optical imaging of tissue can be carried out non-invasively in real time, giving high spatial resolution (<1 μm lateral resolution). A number of optical techniques have been established including confocal microscopy (White et al. 1999; Collier et al. 2005), multispectral fluorescence imaging (Andersson-Engels et al. 1997; Ferris et al. 2001), reflectance spectroscopy with polarised and unpolarised light (Sokolov et al. 1999, 2002; Utzinger et al. 2001), multispectral reflectance imaging with polarised and unpolarised light (Ferris et al. 2001; Gurjar et al. 2001), and fluorescence spectroscopy (Gillenwater et al. 1998; Wagnières et al. 1998; Ramanujam, 2000; Sokolov et al. 2002). Together with emerging molecular tools (e.g. DNA screening, tissue proteomic and serum markers), biomarker imaging may soon be used for real-time screening, diagnosis, and detection of disease recurrence and progression (Rudin and Weissleder, 2003).

Contrast agents consist of a biomarker specific probe molecule, such as an antibody, conjugated to an optically suitable label. By topically applying molecular specific contrast agents to tissues, the scope of molecular changes that can be probed using optical imaging is significantly enhanced. Presently, contrast agents based on metal nanoparticles, organic fluorescent dyes, and quantum dots coupled to monoclonal antibodies against cancer specific biomarkers are being developed (Sokolov et al. 2003; Rahman et al. 2005).

## SAGE Libraries and Tag Mapping

The SAGE technique is capable of producing a molecular representation of cervical tissue based on expressed genes. SAGE is not dependent on pre-existing databases of expressed genes and so provides an independent view of gene expression profiles within the mRNA populations (Velculescu et al. 1997). SAGE library construction is well documented in the literature (Velculescu et al. 1995 and 1997; Madden et al. 2000; Saha et al. 2002; Pleasance et al. 2003; Sander et al. 2005). Several recent gene expression profiles of *in vitro* HPV-infected cultured keratinocytes and from cervical carcinoma clinical samples have proposed changes in gene expression induced by HPV and in early cervical carcinomas (Thomas et al. 2001; Ruutu et al. 2002; Duffy et al. 2003; Pérez-Plasencia et al. 2005). Some studies have compared normal versus tumor-induced gene expression in cervical samples with the aim of identifying potential tumor markers of clinical value (Shim et al. 1998; Chen et al. 2003).

To identify genes expressed at dissimilar levels in preinvasive neoplastic and non-neoplastic untyped cervical tissue, we analysed sixteen long-SAGE libraries; 4 from normal cervical tissue samples, 3 of a mild dysplasia (CINI), 3 of moderate dysplasia (CINII), and 6 of severe dysplasia (CINIII), or carcinoma-in-situ. The CIN tissues are positive for MUC16. Raw numbers of longSAGE tags generated and library names are given in Tables 1 and 2. DiscoverySpace (Robertson et al. 2007), an in-house graphical software application backed by a relational database system designed to support SAGE gene expression analysis, was used to query data from over 25 publicly available data sources, as well as internal experimental results. Using DiscoverySpace, selected SAGE tag sequences were mapped to counterpart RefSeq (Pruitt et al. 2000, 2005) genes and confirmed using SAGE tag co-ordinates to establish gene identity through Ensembl (Hubbard et al. 2007; homo_sapiens_ core_41_36c). Genes were manually curated (EntrezGene) to ascertain gene identity and gene product localisation. These cervical longSAGE libraries were created from the epithelium of cervical biopsy samples collected just prior to LEEP (Loop Electrosurgical Excision Procedure). Tissue samples were placed into RNAIater and frozen at −80 °C within 10 minutes of being excised from the patient. These longSAGE libraries (Shadeo et al. 2007) have been submitted to the NCBI Gene Expression Omnibus (GEO) repository.

Any protein differentially expressed in cancer tissue, compared to normal tissue, or any protein known to be involved in cancer development, has potential as a candidate cancer biomarker. Genes presenting properties which identify them as likely targets for cancer diagnosis or prognosis must be separated from thousands of other genes which also may also possess clinical potential. Hundreds of potential candidates must be set aside in favour of gene products which offer the most promising characteristics. We focus on genes encoding membrane associated proteins because membrane-bound proteins are most likely to be accessible to topical application of contrast agents and have a rapid time frame for contrast agent visualization. Genes expressing the greatest number of tags combined with high levels of differential expression between dysplastic and normal tissue are the most likely to be observed by contrast agents *in vivo*.

For optical imaging of tumors by topical application *in vivo* of contrast agents to be of practical use, a large number of contrast agent receptors are required. One of the standard methods to detect candidate biomarkers is to identify genes with amplified expression in cancer and/or normal tissues. We compared transcription profiles and retained the most highly expressed membrane-bound gene products whose differential expression level is greater than two-fold. Many cell-surface proteins can potentially be developed as targets for optical contrast agents. Using longSAGE, we have also identified the most highly differentially expressed transcripts between disease and normal tissue. Table 1 specifies those genes (with cell-surface gene products) up-regulated in the CINI and CINIII stages of dysplasia and Table 2 lists genes up-regulated in normal tissue. Short descriptions of these protein biomarkers are given in the appendix and annotations of protein structural information, if available, are included. Given that CINII is difficult to determine clinically, it was not included in these comparisons. Contrast agent visualization of the epidermal growth factor receptor (EGFR) using an anti-EGFR monoclonal antibody has already been successful (Rahman et al. 2005). More of these markers should prove amenable to contrast agent development and topical formulations consisting of a range of contrast agents could help adjust to individual patient differences in gene expression.

## Cervical Intraepithelial Neoplasia Stage Biomarkers

It is possible to evaluate a marker for presence or absence, but to correlate a marker or array of markers to changes in cellular localization relative to other markers is probably the most interesting and beneficial in terms of dysplasia progression, environment, therapy selection, and follow-up. The known function of these genes grants some insight into the biology of cervical neoplasia. For instance, several of these cell surface markers are involved in transport and/or signaling. MUCX and CD74, upregulated in CINI and CINIII, have signaling gene products known to be associated with carcinomas. CD74 is also known to be a high affinity binding protein for macrophage migration-inhibitory factor (MIF) which is implicated in tumor cell growth and angiogenesis. TSPAN1, upregulated in CINIII almost 10-fold, also plays a role in cell motility and growth. See Appendix for gene-specific references.

Our analysis of cervical cancer longSAGE expression profiles direct attention to some genes with relatively equal distribution in CINI and CINIII, such as PIGR. Another marker, ANPEP, is present at significantly different expression levels in CINI and CINIII. This knowledge expands the possibilities for rapid visualization between normal and stages of dysplasia *in vivo*. As discussed earlier, these cell surface targets were found by identifying differentially expressed genes. More often than not, a highly expressed tag is not localised to the cell surface and, for these purposes, does not warrant further attention. However, a highly differentially expressed gene whose gene product is not membrane-bound is sometimes found to be part of a mechanism which affects the cell surface and thereby the gene product becomes of potential use. TFF3 (trefoil factor 3), for instance, is up-regulated 13-fold in CINIII and 27-fold in CINI. Members of the trefoil family are characterised by having at least one copy of the trefoil motif, a 40-amino acid domain that contains three conserved disulphides. They are stable, secretory proteins whose functions are not defined but may protect the mucosa from insults, stabilize the mucus layer and affect healing of the epithelium. VANGL1 (Van Gogh-like protein 1) is an integral membrane protein which is serine/threonine phosphorylated and translocated to cytoplasmic vesicles in response to TFF3 stimulation (Kalabis et al. 2006). VANGL1 protein acts as a downstream effector of TFF3 signalling and regulates wound healing of intestinal epithelium. TFF3 is commonly expressed in hepatocellular carcinoma and its expression correlates with tumor grade (Khoury et al. 2005). TFF3 overexpression may be a critical process in mouse and human hepatocellular carcinogenesis (Okada et al. 2005). The group of trefoil factor peptides (TFF1-3) are part of the protective mechanism operating in the intestinal mucosa and play a fundamental role in epithelial protection, repair, and restitution (Vieten et al. 2005). TFF3 and the essential tumor angiogenesis regulator VEGF exert potent pro-invasive activity through STAT3 signalling in human colorectal cancer cells (Rivat et al. 2005). That VANGL1 returned to cell membranes within 45 minutes of TFF3 stimulation (Kalabis et al. 2006) could explain the low VANGL1 tag counts, 1–3 tags per library, observed in the longSAGE libraries.

## Conclusions

Molecular specific contrast agents may provide the ability to directly image the cancer process; but biomarker discovery can be a lengthy process as candidate markers suitable for the task-at-hand must be identified from among thousands of proteins. SAGE-identified biomarkers hold promise for recognition of the stages of neoplasia by proteomic patterns. Optical contrast agents bound to these membrane-bound protein biomarkers will serve as a complement to histopathology, thus allowing more effective determination of tumor borders and non-invasive observation of response to treatment at a molecular level. We present fifty-six cell-surface gene products differentially expressed during progression of cervical intraepithelial neoplasia. Differential gene expression of these biomarkers will allow individualized selection of therapeutic combinations that best target the entire disease-protein system and advance understanding of carcinogenesis.

## Figures and Tables

**Table 1:** Highly expressed genes with membrane-bound gene products up-regulated in cervical dysplasia stages CINI and CINIII. Up-regulated gene expression, from normal, ≥2-fold.

Tag sequence	Number of tags in disease libraries*	Number of tags in normal libraries	Gene	Fold increase from normal	RefSeq accession number
**CINIII**					
GACCCAAGATAAAAGAA	704	16	PIGR	22.4	NM_002644
ATCCCCCTGGGCATCGG	52	2	SLC39A3	13.2	NM_144564
ACTCAGACCAGGTCCCA	94	4	STRA6	11.4	NM_022369
ACACAGTATTCGCTCTT	44	2	ITR	11.2	NM_180989
TTACTTCCCCACCCCTA	84	4	FADS2	10.7	NM_004265
GGAACTGTGAAGAGGCA	230	12	TSPAN1	9.7	NM_005727
GCACCTGTCGCCCAGTG	36	2	ANPEP	9.2	NM_001150
CCTGATCTGCGGTGTCC	308	18	MUC16	8.7	NM_024690
CGTTTTCTGATAACTCA	68	4	PTP4A2	8.6	XM_001132367
CAAATAAATTATGCGAT	98	6	TMPRSS2	8.3	NM_005656
TGCTCCTACCCTGCTCT	2022	190	FCGBP	5.4	XM_001131379
GCAGTGCCACTCAAGAA	726	68	SRD5A2L	5.4	NM_024592
CCTGGGAAGTGTTGTGG	730	98	MUC1	3.8	NM_001044391
AATATTTATATTGTATG	1880	400	CEACAM5	2.4	NM_004363
GTTCACATTAGAATAAA	3424	762	CD74	2.3	NM_001025158
GGGCATCTCTTGTGTAC	1602	350	HLA-DRA	2.3	NM_019111
TGCTGCCTGTTGTTATG	826	192	BST2	2.2	NM_004335
CTGACCTGTGTTTCCTC	596	144	HLA-B	2.1	NM_005514
GCAGGGCCTCATCTCAC	1950	484	FXYD3	2.0	NM_005971

**CINI**					
GCACCTGTCGCCCAGTG	42	2	ANPEP	36.5	NM_001150
ATGTTAATAAAATAGGC	66	4	GPC4	28.7	NM_001448
ATAAATGATTAGACTAC	32	2	CLIC6	27.8	NM_053277
GGCCAAGAACTTTCACT	28	2	C14orf101	24.3	NM_017799
GACCCAAGATAAAAGAA	262	16	PIGR	23.0	NM_002644
TAGGTCAGGACCTTGGC	26	2	PTP4A3	22.6	NM_032611
GTATATAACTCTTAAAG	24	2	LOC644410**	20.8	XM_001133198
CCAGCTGCCTGGAGGAG	22	2	MGC45438	19.1	NM_152459
ATAAAAATTAGGGGGAT	22	2	SLC30A1	19.1	NM_021194
TGAGCTACCCCAGAGTC	20	2	FER1L4	17.4	NR_001442
TACATCAGTAAAGAGTT	374	42	GAS1	12.5	NM_002048
CATCACGGATCAATAGA	330	64	IL1R1	7.2	NM_000877
CCTGGGAAGTGTTGTGG	334	98	MUC1	4.1	NM_001044391
TGCAGATTGCAGTTCTG	366	132	PROM1	3.9	NM_006017
CACTTCAAGGGCAGCCT	238	102	LY6E	3.3	NM_002346
GTTCACATTAGAATAAA	1660	762	CD74	3.1	NM_001025158
GGGCATCTCTTGTGTAC	734	350	HLA-DRA	3.0	NM_019111
TGCTCCTACCCTGCTCT	402	190	FCGBP	3.0	XM_001131379
CTGACCTGTGTTTCCTC	256	144	HLA-B	2.5	NM_005514

* GEO Series Accession Numbers GSE6252. 1,101,702 total longSAGE tags in four normal libraries, GEO Alias N3, N1, N2, N4; 2,165,777 total longSAGE tags in six CINIII libraries, GEO Alias C1, C3, C2, C4, C5, C6; 785,642 total longSAGE tags in three CINI libraries, GEO Alias M1, M2, M3.

** Mapping unable to be confirmed through ENSEMBL or BLAT.

**Table 2:** Highly expressed genes with membrane-bound gene products up-regulated in normal cervical tissue. Up-regulated gene expression, from CINI and/or CINIII, ≥2-fold.

Tag sequence	Number of tags in disease libraries*	Number of tags in normal libraries	Gene	Fold increase from disease state	RefSeq accession number
**CINIII**					
AGACTTGGCATACACAC	2	30	ACTR3	29.5	NM_005721
TAACTGGCCTTACGATG	6	54	DSG1	17.7	NM_001942
AGAGGGATGAGGCAACC	2	18	GJB2	17.7	NM_004004
AGTCCTGGAGGAGGAGA	2	18	PTPNS1	17.7	NM_001040022
GCTGACAGTCCCAAGTC	2	16	NIP	15.7	NM_144565
ACAGTCACCACGAGGAG	2	16	ODZ2	15.7	XM_047995
AGAGTTCTGTCACGGTC	8	62	UPK1A	15.2	NM_007000
CAGTATTTTAAATAATT	2	12	FLJ32028	11.8	NM_152680
ATAATCACCCTTCATCA	2	12	CACNA2D3	11.8	NM_018398
TGGTGGCTGCTTTTCCC	2	10	TMEM54	9.8	NM_033504
CCAGCGCCAACCAGTCA	272	482	LYPD3	3.5	NM_014400
GTTTCCAAAAAATGGTA	350	438	GJB2	2.5	NM_004004
GTGGAAGACGATAACCC	5664	7334	MAL	2.5	NM_002371
GCCCAGCATTCTCCACC	856	832	PSCA	1.9	NM_005672
CTTGATTCCCACGCTAC	236	230	QSCN6	1.9	NM_002826
GTTATTGAGGGCAAGAA	196	194	C3orf28	1.9	NM_014367

**CINI**					
CAATCTTGCAGTGAAGA	6	226	TMPRSS11B	26.9	NM_182502
TCAGAATGATTCTGGTG	22	346		11.2	
AGAGTTCTGTCACGGTC	2	62	UPK1A	22.1	NM_007000
GCATAACAACTCCCCAA	4	60	EMP1	10.7	NM_001423
TAATTTGCATTACTCTG	366	2036		4.0	
GATCCAGATGCCTGAGG	4	74	LYNX1	13.2	NM_023946
CCAGCGCCAACCAGTCA	28	482	LYPD3	12.3	NM_014400
GATGAATCCGGGGTATG	4	68	TMEM45B	12.1	NM_138788
TATATTTTTAAATGTTC	2	30	TGFA	10.7	NM_003236
TAACCCCTCTCACCTGC	2	28	FGFR2	10.0	NM_022970
GGGACTAAAACTCACCC	2	28	MAL	10.0	NM_002371
GGAGGGAGCTGAGGGGT	2	28	EPHA1	10.0	NM_005232
GCCCAGCATTCTCCACC	82	832	PSCA	7.2	NM_005672
GTTATTGAGGGCAAGAA	30	194	C3orf28	4.6	NM_014367
GTTTCCAAAAAATGGTA	70	438	GJB2	4.5	NM_004004
GCTTCCTCGGTACCCTT	252	1590	RHCG	4.5	NM_016321
CCACAGGAGAATTCGGG	952	5624	PERP	4.2	NM_022121
GATATGTAAAAACTGTC	38	194	CLCA4	3.6	NM_012128
GCCTACCCGAGGAGAAG	132	616	TACSTD2	3.3	NM_002353

* GEO Series Accession Number GSE6252. 1,101,702 total longSAGE tags in four normal libraries, GEO Alias N3, N1, N2, N4; 2,165,777 total longSAGE tags in six CINIII libraries, GEO Alias C1, C3, C2, C4, C5, C6; 785,642 total longSAGE tags in three CINI libraries, GEO Alias M1, M2, M3.

## Appendix

### Biomarker descriptions

ACTR3 ARP3 actin-related protein 3 homolog (yeast). The protein encoded by this gene is known to be a major constituent of the ARP2/3 complex (Welch et al. 1997). This complex is located at the cell surface and is essential to cell shape and motility through lamellipodial actin assembly and protrusion (Machesky et al. 1997).

Structure information: 1K8K Crystal Structure of Arp2/3 Complex (Robinson et al. 2001).

*ANPEP* alanyl (membrane) aminopeptidase (aminopeptidase N, aminopeptidase M, microsomal aminopeptidase, CD13, p150). Human aminopeptidase N is a receptor for one strain of human coronavirus (Yeager et al. 1992; Breslin et al. 2003). The large extra-cellular carboxyterminal domain contains a pentapeptide consensus sequence characteristic of members of the zinc-binding metalloproteinase super-family. Defects in this gene appear to be a cause of various types of leukemia or lymphoma.

*BST2* bone marrow stromal cell antigen 2. Bone marrow stromal cells are involved in the growth and development of B-cells and this protein may play a role in pre-B-cell growth (Ishikawa et al. 1995).

*C3orf28* chromosome 3 open reading frame 28 (HGTD-P). When overexpressed, HGTD-P induces cell death via mitochondrial apoptotic cascades (Lee et al. 2004; Kim et al. 2006).

*C14orf101* chromosome 14 open reading frame 101. Integral to membrane (Ensembl-Gene ENSG00000070269: inferred from electronic annotation).

*CACNA2D3* calcium channel, voltage-dependent, alpha 2/delta 3 subunit. This gene encodes a member of the alpha-2/delta subunit family, a protein in the voltage-dependent calcium channel complex (Hanke et al. 2001).

*CD74 CD74* molecule, major histocompatibility complex MHC, class II invariant chain. CD74 is a nonpolymorphic type II integral membrane protein with an N-terminal cytoplasmic tail. CD74 is a regulated intra-membrane proteolysis RIP protein and its roles as a chaperone and as a signalling molecule are tightly regulated (Stumptner-Cuvelette and Benaroch, 2002; Becker-Herman et al. 2005). In addition, CD74 is an accessory signalling molecule during T-cell responses through interactions with CD44 (Naujokas et al. 1993). Providing further evidence for its role in signal transduction pathways, CD74 is now known to be a high affinity binding protein for the proinflammatory cytokine, macrophage migration-inhibitory factor (MIF); MIF binds to the extracellular domain of CD74 (Leng et al. 2003). MIF is also implicated in tumour cell growth and angiogenesis (Nishihira, et al. 2003).

Structure information: 1IIE (75 amino acids; 296 in CD74) HLA-DR antigens associated invariant chain (Jasanoff et al. 1998). 1MIF Macrophase migration inhibitory factor (MIF) (Sun et al. 1996).

*CEACAM5* carcinoembryonic antigen-related cell adhesion molecule 5. CEACAM1 interacts heterophilically with the CEA (CEACAM5) protein. Because CEA is expressed on a wide range of carcinomas and commonly used as tumour marker, a novel role for the CEA protein enabling the escape of tumour cells from NK-mediated killing is now apparent (Stern et al. 2005).

Structure information: 1E07 Model of human carcinoembryonic antigen by homology modelling and curve-fitting to experimental solution scattering data (Boehm and Perkins, 2000). 2GK2 Crystal structure of the N-terminal domain of human CEACAM1 (Fedarovich et al. 2006). Using the BLOSUM62 comparison matrix, CEACAM1 (468 residues) has 74.3% identity to CEACAM5 (702 residues) in 412 residue overlap.

*CLCA4* chloride channel, calcium activated, family member 4. The protein encoded by this gene belongs to the calcium sensitive chloride conductance protein family; the exact function is not known (Agnel et al. 1999; Ritzka et al. 2004).

*CLIC6* chloride intracellular channel 6. This gene encodes a member of the chloride intracellular channel family of proteins.

Structure information: 2D2Z Crystal structure of soluble form of CLIC4 (Li et al. 2006).

*DSG1* desmoglein 1. Desmoglein 1 is a calcium-binding trans-membrane glycoprotein component of desmosomes in vertebrate epithelial cells (Wheeler et al. 1991; Hanakawa et al. 2003).

*EMP1* epithelial membrane protein 1. A well-known tumour-associated gene and a member of a novel family of genes encoding membrane glycoproteins (Ben-Porath and Benvenisty, 1996; Schiemann et al. 1997), EMP1 is a biomarker of gefitinib resistance (Jain et al. 2005). Gefitinib is a small-molecule inhibitor that competes for the ATP-binding site on EGF receptor (EGFR) and has been approved for patients with advanced lung cancers.

Structure information: 1EBP Complex between the extracellular domain of erythropoietin (EPO) receptor [EBP] and an agonist peptide [EMP1] (Livnah et al. 1996).

*EPHA1* EPH receptor A1. EPH and EPH-related receptors have been implicated in mediating developmental events, particularly in the nervous system (Flanagan and Vanderhaeghen, 1998). Structure information: 2GSF The Human Epha3 Receptor Tyrosine Kinase and Juxtamembrane Region (Davis et al. 2006). 1MQB Crystal Structure of Ephrin A2 (ephA2) Receptor Protein Kinase (Nowakowski et al. 2003).

*FADS2* fatty acid desaturase 2. FADS family members are considered fusion products composed of an N-terminal cytochrome b5-like domain and a C-terminal multiple membrane-spanning desaturase portion, both of which are characterized by conserved histidine motifs (Marquardt et al. 2000; Schaeffer et al. 2006).

*FCGBP* Fc fragment of IgG binding protein (Kobayashi et al. 1991; Harada et al. 1997; O’Donovan et al. 2002). The encoded protein is made up of 3004 amino acid residues.

*FER1L4* fer-1-like 4 (*C. elegans*). Novel human gene OTOF is the second member of a mammalian gene family related to *C. elegans* fer-1. It encodes a predicted cytosolic protein with a single carboxyterminal trans-membrane domain. The sequence homologies and predicted structure of otoferlin, the protein encoded by OTOF, suggest its involvement in vesicle membrane fusion (Yasunaga et al. 1999).

*FGFR2* fibroblast growth factor receptor 2 (bacteria-expressed kinase, keratinocyte growth factor receptor, craniofacial dysostosis 1, Crouzon syndrome, Pfeiffer syndrome, Jackson-Weiss syndrome). FGFR2 is a high-affinity receptor for acidic, basic and/or keratinocyte growth factor, depending on the isoform (Bansal et al. 1997). The extra-cellular portion of the protein interacts with fibroblast growth factors, setting in motion a cascade of downstream signals, ultimately influencing mitogenesis and differentiation.

Structure information: 1OEC FGFR2 kinase domain (Ceska et al. 2004).

*FLJ32028* transmembrane protein 154. Hypothetical protein LOC201799. Integral to membrane (Ensembl-Gene ENSG00000170006: inferred from electronic annotation).

*FXYD3* FXYD domain containing ion transport regulator 3. FXYD2, also known as the gamma subunit of the Na,K-ATPase, regulates the properties of that enzyme. Trans-membrane topology has been established for two family members (FXYD1 and FXYD2), with the N-terminus extracellular and the C-terminus on the cytoplasmic side of the membrane (Crowell et al. 2003; Franzin et al. 2005). The protein encoded by this gene may function as a chloride channel or as a chloride channel regulator.

*GAS1* growth arrest-specific 1. GAS1 plays a role in growth suppression and blocks entry to S phase and prevents cycling of normal and transformed cells (Del Sal et al. 1992). Gas1 is a putative tumour suppressor gene (Evdokiou and Cowled, 1998).

*GJB2* gap junction protein, beta 2, 26kDa. The cytoplasmic domains of the connexion 26 gap junction surface, imaged at sub-molecular resolution, form a hexameric pore protruding from the membrane bilayer (Muller et al. 2002).

*GPC4* glypican 4. Cell surface heparan sulfate proteoglycans are composed of a membrane-associated protein core substituted with a variable number of heparan sulfate chains. These proteins may play a role in the control of cell division and growth regulation (Bernfield et al. 1999).

*HLA-B* major histocompatibility complex, class I, B. HLA-B belongs to the HLA class I heavy chain paralogues. Class I molecules play a central role in the immune system by presenting peptides derived from the endoplasmic reticulum lumen and are expressed in nearly all cells. The heavy chain is anchored in the membrane.

Structure information: 1HSA The three-dimensional structure of HLA-B27 at 2.1 Å resolution suggests a general mechanism for tight peptide binding to MHC (Madden et al. 1992).

*HLA-DRA* major histocompatibility complex, class II, DR alpha. It is a heterodimer consisting of an alpha and a beta chain, both anchored in the membrane, and it plays a central role in the immune system.

Structure information: 1AQD HLA-DR1 (DRA, DRB1 0101) Human class II histocompatibility protein (extracellular domain) complexed with endogenous (Murthy and Stern, 1997). 1YMM TCR/HLA-DR2b/MBP-peptide complex (Hahn et al. 2005).

*IL1R1* interleukin 1 receptor, type I. This protein is a receptor for interleukin alpha, interleukin beta, and interleukin 1 receptor, type I (Dower et al. 1986; McMahan et al. 1991). It is an important mediator involved in many cytokine induced immune and inflammatory responses (Boch et al. 2003). Structure information: 1IRA Complex of the interleukin-1 receptor with the interleukin-1 receptor antagonist (IL1RA) (Schreuder et al. 1997).

*ITR G* protein-coupled receptor 180. This protein is produced predominantly in vascular smooth muscle cells and may play an important role in the regulation of vascular remodelling (Iida et al. 2003).

*LOC644410* FCGR1C Fc fragment of IgG, high affinity Ic, receptor (CD64). Only Fc gamma RI has high affinity for ligand and has a unique third extra-cellular domain (EC3). Three genes for human Fc gamma RI (A, B, and C) have been characterised; although they are remarkably similar, genes B and C are notably different from A (Ernst et al. 1992).

Structure information: 1E4J Crystal structure of the soluble human FC-gamma receptor III (Sondermann et al. 2000).

*LY6E* lymphocyte antigen 6 complex, locus E. Acute promyelocytic leukemia APL is a human malignancy that responds to differentiation therapy with all-trans-retinoic acid ATRA (Huang et al. 1988). ATRA induces the expression of a novel human gene, RIG-E (Mao et al. 1996). The amino acid composition of its product indicates that it is membrane-associated and has high homology to the murine LY-6 proteins and weak homology with a number of human growth factor receptors.

*MGC45438* hypothetical protein MGC45438. Type I membrane protein (LOCATE).

*LYNX1* Ly6/neurotoxin 1. Ly-6/neurotoxin gene family members are lymphocyte antigens that attach to the cell surface by a glycosylphosphatidylinositol anchor and have a unique structure displayed 8–10 conserved cysteine residues (Tsuji et al. 2003). Functional analysis indicates that LYNX1 can enhance nicotinic acetylcholine receptor function in the presence of acetylcholine (Arredondo et al. 2006). It is a new marker for human breast cancer (Lee et al. 2006).

*LYPD3* LY6/PLAUR domain containing 3 (C4.4A). This protein is known to be a structural homologue of the urokinase-type plasminogen activator receptor (uPAR) but little is known about its function (Hansen et al. 2004).

*MAL* mal, T-cell differentiation protein. The protein encoded by this gene is a highly hydrophobic integral membrane protein belonging to the MAL family of proteolipids (Llorente et al. 2004; Dukhovny et al. 2006).

*MUC1* mucin 1, cell-surface associated. This gene is a member of the mucin family and encodes a membrane bound, glycosylated phosphoprotein. The protein is anchored to the apical surface of many epithelia by a trans-membrane domain, with the degree of glycosylation varying with cell type. The protein serves a protective function by binding to pathogens and also functions in a cell signalling capacity (Ren et al. 2006). Over-expression, aberrant intracellular localization, and changes in glycosylation of this protein have been associated with carcinomas (Rabassa et al. 2006; Raina et al. 2006).

Structure information: 2ACM Solution structure of the SEA domain of human mucin 1 (MUC1) (Macao et al. 2006).

*MUC16* mucin 16 (CA125), cell surface associated. CA125 protein core is composed of a short cytoplasmic tail, a trans-membrane domain, and an extraordinarily large glycosylated extracellular structure. The extracellular domain encompasses an interactive disulfide bridged cysteine-loop and the site of OC125 and M11 binding (O’Brien et al. 2001). It is known to be a marker in several cancers, including ovarian (Yin et al. 2002), renal (Bamias et al. 2003), and lung (Pollan et al. 2003).

*NIP* hypothetical protein FLJ32334 (DUOXA1). Multi-pass membrane protein (Ensembl-Gene ENSG00000140254: inferred from electronic annotation).

*ODZ2* odz, odd Oz/ten-m homolog 2 (Drosophila). This protein is membrane-bound transcription regulator (Baqutti et al. 2003).

*PERP* PERP, TP53 apoptosis effector. This tetraspan protein localizes to the plasma membrane, rather than to mitochondria, and may stimulate apoptosis (Ihrie and Attardi, 2004).

*PIGR* polymeric immunoglobulin receptor. PIGR mediates trans-cellular transport of polymeric immunoglobulin molecules. The receptor has 5 units with homology to the variable (V) units of immunoglobulins and a trans-membrane region, which also has some homology to certain immunoglobulin variable regions.

Structure information: 1XED Crystal Structure of a Ligand-Binding Domain of the Human Polymeric Ig Receptor, pIgR (Hamburger et al. 2004).

*PROM1* prominin 1. The PROM1 gene codes for a pentaspan trans-membrane glycoprotein. The PROM1 antigen appears to belong to a new molecular family of 5-TM proteins which include an extra-cellular N-terminus, two short intracellular loops, two large extra-cellular loops and an intra-cellular C-terminus. PROM1 has been shown to be expressed on haemangioblasts and neural stem cells as well as on developing epithelium (Horn et al. 1999; Florek et al. 2005). No natural ligand has yet been demonstrated for the PROM1 molecule (Wu et al. 2006).

*PSCA* prostate stem cell antigen. This gene encodes a glycosylphosphatidylinositol-anchored cell membrane glycoprotein and a target for immunotherapy (Reiter et al. 1998; Wente et al. 2005).

*PTP4A2* protein tyrosine phosphatase type IVA, member 2. The protein encoded by this gene belongs to a small class of prenylated protein tyrosine phosphatases (PTPs). PTPs are cell signalling molecules that play regulatory roles in a variety of cellular processes (Bardelli et al. 2003; Rouleau et al. 2006). Overexpression of this gene in mammalian cells conferred a transformed phenotype, which suggested its role in tumourigenesis (Cates et al. 1996).

Structure information: 1XM2 Crystal structure of Human PRL-1 (Jeong et al. 2005).

*PTP4A3* protein tyrosine phosphatase type IVA, member 3. Over-expression of this PTP gene in mammalian cells was reported to inhibit angiotensin-II induced cell calcium mobilization and promote cell growth (Matter et al. 2001).

Structure information: *1V3A* Structure of human PRL-3, the phosphatase associated with cancer metastasis (Kim et al. 2004).

*PTPNS1* SIRPA signal-regulatory protein alpha. Signal-regulatory-protein (SIRP) family members are receptor-type transmembrane glycoproteins known to be involved in the negative regulation of receptor tyrosine kinase-coupled signalling processes (Kharitonenkov et al. 1997). CD47 has been known to be a ligand for this PTPNS1 (Subramanian et al. 2006).

Structure information: 2D9C Solution structure of the first ig-like domain of signal-regulatory protein beta-1 (SIRP-beta-1) (Nagashima et al. 2005).

*RHCG* Rh family, C glycoprotein. RhCG facilitates ammonium movement across the plasma membrane (Zidi-Yahiaoui et al. 2005).

*SLC30A1* solute carrier family 30 (zinc transporter), member 1. Transports zinc out of cells; its absence accounts for increased sensitivity of mutant cells to zinc toxicity (Palmiter and Findley, 1995).

*SLC39A3* solute carrier family 39 (zinc transporter), member 3. These eight-transmembrane domain proteins are part of the Zrt/Irt-like protein (ZIP) super-family of metal ion transporters and contain a conserved 12-amino acid signature sequence within the fourth transmembrane domain (Gaither and Eide, 2000, 2001; Dufner-Beattie et al. 2003).

*SRD5A2L* steroid 5 alpha-reductase 2-like; 3-oxo-5-alpha-steroid 4 dehydrogenase activity.

*STRA6* stimulated by retinoic acid gene 6 homolog (mouse). Stra6 codes for a very hydrophobic membrane protein of a new type, which does not display similarities with previously characterized integral membrane proteins (Bouillet et al. 1997).

*TACSTD2* tumour-associated calcium signal transducer 2. This gene encodes a carcinoma-associated antigen, defined by the monoclonal antibody GA733. TACSTD2 transduces an intra-cellular calcium signal and acts as a cell surface receptor (Ripani et al. 1998).

*TFF3* trefoil factor 3. Trefoil family members are stable, secretory proteins having at least one copy of the trefoil motif, a 40-amino acid domain that contains three conserved disulphides. VANGL, Van Gogh-like protein 1, is phosphorylated in response to Intestinal Trefoil Factor (ITF) stimulation. Vangl1 protein acts as a downstream effector of ITF/TFF3 signalling and regulates wound healing of the intestinal epithelium (Kalabis et al. 2006). TFF3 is commonly expressed in hepatocellular carcinoma and its expression correlates with tumour grade (Khoury et al. 2005).

Structure information: 1E9T High resolution solution structure of human intestinal trefoil factor (Lemercinier et al. 2001; Muskett et al. 2003).

*TGFA* transforming growth factor, alpha. TGF-alpha shows about 40% sequence homology with epidermal growth factor (EGF) and competes with EGF for binding to the EGF receptor (Lee et al. 1985; Winkler et al. 1989).

Structure information: 1MOX Crystal Structure of Human Epidermal Growth Factor Receptor (residues 1-501) in complex with TGF-alpha (Garret et al. 2002). 2TGF The solution structure of human transforming growth factor alpha (Harvey et al. 2001).

*TMEM45B* transmembrane protein 45B. Integral to membrane (Ensembl-Gene ENSG0000051715: inferred from electronic annotation).

*TMEM54* transmembrane protein 54. Integral to membrane (Ensembl-Gene ENSG00000121900: inferred from electronic annotation).

*TMPRSS2* transmembrane protease, serine 2. This gene was demonstrated to be up-regulated by androgenic hormones in prostate cancer cells and down-regulated in androgen-independent prostate cancer tissue (Afar et al. 2001). The protease domain of this protein is thought to be cleaved and secreted into cell media after autocleavage.

The encoded protein contains a type II transmembrane domain, a receptor class A domain, a scavenger receptor cysteine-rich domain and a protease domain.

Structure information: 1Z8G Crystal structure of the extracellular region of the transmembrane serine protease hepsin with covalently bound preferred substrate (Herter et al. 2005).

*TMPRSS11B* transmembrane protease, serine 11B. Integral to membrane (Ensembl-Gene ENSG00000185873: inferred from electronic annotation).

Structure information: 1Z8G (see TMPRSS2)

*TSPAN1* tetraspanin 1. Most members of the trans-membrane 4 superfamily are cell-surface proteins characterized by the presence of four hydrophobic domains. The proteins mediate signal transduction events that play a role in the regulation of cell development, activation, growth and motility (Todd et al. 1998). Tetraspanin protein, C4.8, identical to NET-1, has been implicated in cervical carcinogenesis (Wollscheid et al. 2002).

*UPK1*A uroplakin 1A. The tetraspanin protein encoded by this gene is found in the asymmetrical unit membrane (AUM) where it can complex with other trans-membrane 4 superfamily proteins (Yu et al. 1994). It may play a role in regulating membrane permeability of superficial umbrella cells or in stabilizing the apical membrane through AUM/cytoskeletal interactions (Riedel et al. 2005).
